# Ziv-Aflibercept Use in Metastatic Colorectal Cancer

**DOI:** 10.6004/jadpro.2013.4.5.6

**Published:** 2013-09-01

**Authors:** Mabel Rodriguez

**Affiliations:** From Memorial Sloan-Kettering Cancer Center, New York, New York

Colorectal cancer (CRC) is the third most common cancer in both men and women (Siegel, Naishadham, & Jemal, 2013). In 2011, the global number of newly diagnosed cases worldwide was more than 1 million, with 600,000 deaths worldwide (Jemal et al., 2011). Although incidence and mortality rates in the United States have decreased over time, it is estimated that 102,480 new cases of CRC will be diagnosed in 2013, with 50,830 deaths expected (Siegel et al., 2013).

Most cases of CRC arise sporadically; however, some risk factors include age, previous colonic polyps, and inflammatory bowel disease. Environmental factors such as consuming red meat, maintaining a high-fat diet, and smoking can also predispose an individual to CRC. Hereditary syndromes such as Lynch syndrome and familial adenomatous polyposis are also associated with the development of CRC (Cunningham et al., 2010).

Approximately 60% of CRC patients are diagnosed in the locally advanced or metastatic disease stage, with the latter carrying a poor prognosis with approximately a 10% 5-year overall survival rate (Wang & Lockhart, 2012). In the past 5 years, the main advancement in the treatment of metastatic colorectal cancer (mCRC) was the addition of targeted treatments to palliative chemotherapy. The epidermal growth factor receptor (EGFR)-directed monoclonal antibodies cetuximab (Erbitux) and panitumumab (Vectibix) have shown activity in patients with wild-type KRAS CRC. Bevacizumab (Avastin) is an antiangiogenic humanized monoclonal antibody against vascular endothelial growth factor A (VEGF-A), which by normalization of tumor vasculature can improve drug delivery to the tumor (Cunningham et al., 2010).

## Angiogenesis

Angiogenesis, a complex process that results in the growth of new blood vessels from preexisting vasculature, is a key step in cancer development and progression. Neoplastic cells secrete proangiogenic factors that stimulate endothelial cell division and migration. The increase in blood vessels is a consequence of angiogenic proteins that include VEGF, placental growth factors, and platelet-derived growth factors, among others. These ligands interact extracellularly with their receptors, causing activation of downstream pathways that leads to vascular cell proliferation, differentiation, and migration (Ferrarotto & Hoff, 2012).

## Pharmacology and Pharmacokinetics

Ziv-aflibercept (Zaltrap) is a soluble recombinant fusion protein that was developed by fusing sections of the second immunoglobulin (Ig) domain to VEGFR-1 and the third Ig domain of VEGFR-2 to the Fc portion of human IgG1. These soluble receptors bind to VEGF-A, VEGF-B, and PlGF (placental growth factor) ligands with high affinity; ziv-aflibercept functions as a decoy, preventing their binding to native receptors and furthermore inhibiting angiogenesis (Sanofi-Aventis, 2012; Wang & Lockhart, 2012; Mitchell, 2012). Pharmacokinetic studies measured both free and VEGF-bound plasma concentrations of ziv-aflibercept by enzyme-linked immunosorbent assay (ELISA). Evaluation of doses ranged from 2 to 9 mg/kg, where free ziv-aflibercept concentrations exhibit linear pharmacokinetics. For the specific US Food and Drug Administration (FDA)-approved dosage of 4 mg/kg every 2 weeks, the elimination half-life of free ziv-aflibercept was approximately 6 days (range, 4–7 days). Steady-state concentrations were achieved by the second dose of the drug, with an accumulation ratio of 1.2 (Sanofi-Aventis, 2012).

These pharmacokinetic parameters do not appear to be affected in patients with mild or moderate hepatic impairment. There are no data regarding patients with severe hepatic impairment (total bilirubin > 3 and any SGOT/AST elevation). Likewise, renal dysfunction had no effect on ziv-aflibercept clearance, which was demonstrated by five patients in the population pharmacokinetic analysis whose creatinine clearance was < 30 mL/min. The only factor that appeared to be of relevance was noted in patients weighing > 100 kg, as they experienced a 29% increase in systemic exposure compared to patients weighing between 50 and 100 kg (Sanofi-Aventis, 2012).

## Clinical Development

**PHASE I**

Several phase I trials of single-agent ziv-aflibercept as well as the agent in combination have been completed in solid tumors. The first phase I study published in 2010 by Lockhart and colleagues included 47 patients with a variety of refractory solid tumors, 7 of whom had CRC. Ziv-aflibercept was administered intravenously in escalating doses from 3 to 7 mg/kg every 2 weeks. The dose-limiting toxicities in this study were proteinuria and rectal ulceration, seen at the dose of 7 mg/kg.

Responses were observed in four patients: three with ovarian cancer and one with malignant thymoma. Increased toxicity with higher doses, as well as VEGF saturation at doses ³ 2 mg/kg, determined the recommended dose for phase II and III studies to be 4 mg/kg (Lockhart et al., 2010; Gaya & Tse, 2012; Wang & Lockhart, 2012). A recent regional combination phase I trial in Japanese patients with mCRC also determined the ideal dose for further studies in this patient population to be 4 mg/kg (Yoshino et al., 2012).

**PHASE II**

One multicenter, open-label trial studied 51 patients with previously treated mCRC who were enrolled in one of two cohorts: bevacizumab-naive or treated. The most common adverse reactions were fatigue, hypertension, and proteinuria. It was concluded that ziv-aflibercept had modest activity against refractory/relapsed mCRC regardless of prior therapy with bevacizumab (Tang et al., 2008; Wang & Lockhart, 2012).

Another phase II trial (AFFIRM) evaluated 230 patients with mCRC treated with modified FOLFOX6 (leucovorin, fluorouracil [5-FU], and oxaliplatin) plus aflibercept vs. control as first-line therapy. Preliminary results were disclosed at the 2011 Deutsche Bank BioFEST conference: The progression-free survival (PFS) rate at 1 year was similar to that seen in the standard therapy arm (He et al., 2012; ClinicalTrials.gov, 2013).

**PHASE III**

Several phase III trials have studied the clinical efficacy of ziv-aflibercept in metastatic non–small cell lung cancer (NSCLC) as well as pancreatic, prostate, and colorectal cancers. The VANILLA study included treatment with gemcitabine (Gemzar) plus ziv-aflibercept or placebo in 546 patients with metastatic pancreatic cancer (ClinicalTrials.gov, 2012). The trial was terminated early because it did not show an improvement in overall survival (OS) or PFS.

The VITAL study was conducted in patients with locally advanced or metastatic NSCLC receiving docetaxel plus ziv-aflibercept or docetaxel plus placebo as second-line treatment (Ramlau et al., 2012). This study did not meet its primary endpoint of OS; however, it showed activity with an improved hazard ratio (HR) of 0.82 (95% confidence interval [CI] = 0.716–0.937) and an objective response rate (ORR) of 23.3% (vs. 8.9% in the placebo arm).

VENICE randomized 1224 metastatic androgen-independent prostate cancer patients to receive docetaxel, prednisone, and ziv-aflibercept or docetaxel, prednisone, and placebo. The addition of ziv-aflibercept did not result in improved OS. Higher adverse events such as hypertension, infections, and hemorrhagic events were recorded in the study group (Tannock et al., 2013).

VELOUR, the phase III trial that led to the FDA approval of ziv-aflibercept, compared ziv-aflibercept plus leucovorin, 5-FU, and irinotecan (FOLFIRI) vs. placebo plus FOLFIRI in patients previously treated with an oxaliplatin-based regimen. The goal of this study was to establish statistically improved overall and median survival with the addition of ziv-aflibercept—a VEGF trap and the first of its class—when combined with FOLFIRI in a second-line treatment setting (Van Cutsem et al., 2012).

The VELOUR study was a prospective, randomized, multinational, double-blind parallel study that included 1226 patients who had documented disease progression while on or after completion of a single prior oxaliplatin-containing regimen, regardless of the timing of their progression. Patients who had previously been treated with irinotecan were excluded, but prior bevacizumab use was allowed. Patients were treated with IV ziv-aflibercept 4 mg/kg given over 1 hour on day 1 every 2 weeks, followed immediately by FOLFIRI. If patients permanently discontinued either of the treatment components due to specific toxicities, they were allowed to receive the remaining constituent. Patients in the study arm showed an improved median survival of 13.5 vs. 12.06 months (HR, 0.817; 95% CI = 0.713–0.937;* p* = .0032).

Progression-free survival was also statistically improved: 6.9 vs. 4.7 months (HR, 0.758; 95% CI = 0.661–0.869;* p* = .0001). Dose reduction or omission occurred in 17% of patients on the ziv-aflibercept/FOLFIRI arm compared with 5% on the placebo arm. Cycle delays of 7 days or more also occurred in 60% vs 43%, respectively (Van Cutsem et al., 2012).

## Indication

In August 2012, ziv-aflibercept received FDA approval for use in combination with FOLFIRI in patients with mCRC that is resistant to or has progressed after receiving oxaliplatin-containing regimens such as FOLFOX (Sanofi-Aventis, 2012).

## Dosing and Administration

The manufacturer’s recommended dose is 4 mg/kg given as an IV infusion over 1 hour to be administered every 2 weeks. Ziv-aflibercept should be administered before any of the chemotherapeutic agents contained in the FOLFIRI regimen. Continuation of this therapy must be based on patient tolerability; otherwise, therapy should continue until disease progression is observed (Sanofi-Aventis, 2012).

## Safety

There are no contraindications to the use of ziv-aflibercept; however, precautions with respect to several conditions accompany its endorsement: hemorrhage, gastrointestinal perforation, fistula formation, compromised wound healing, hypertension, arterial thromboembolic events, and proteinuria, among others (Sanofi-Aventis, 2012).

Adverse reactions from the phase III VELOUR study previously discussed were reported. Adverse events led to permanent discontinuation of treatment in 26.8% of the patients in the ziv-aflibercept arm and 12.1% of those in the control arm. Of these, the most common were asthenic conditions, diarrhea, hypertension, neutropenia, infections, stomatitis, and venous thromboembolic events (Van Cutsem et al., 2012).

Similarly, the most common adverse reactions reported (≥ 20% incidence) were leukopenia, diarrhea, proteinuria, stomatitis, fatigue, thrombocytopenia, AST/ALT elevation, hypertension, decreased appetite and weight loss, epistaxis, abdominal pain, dysphonia, and headache. Grade 3/4 adverse events were more commonly reported in the ziv-aflibercept arm: 83.5 % vs. 62.5%. Specifically, there was a higher incidence of hypertension, hemorrhage, thromboembolic events, proteinuria, weight loss, and dysphonia observed in the study arm as compared to the control arm (Table 1).

**Table 1:**
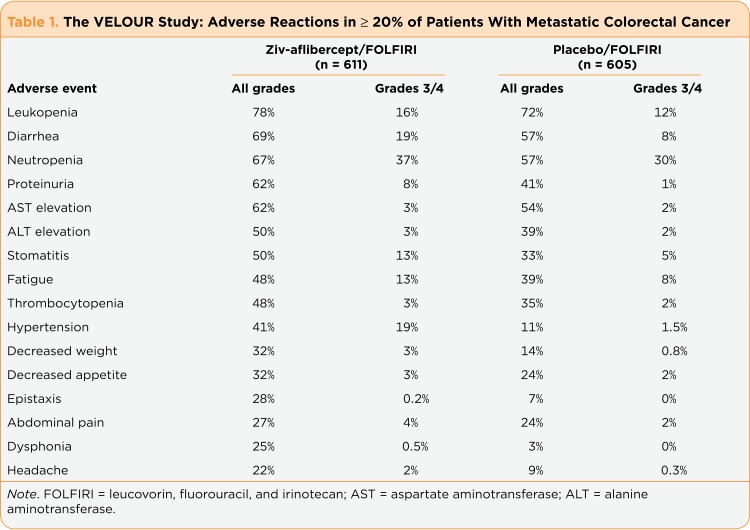
Table 1. The VELOUR Study: Adverse Reactions in ≥ 20% of Patients With Metastatic Colorectal Cancer

A randomized, placebo-controlled trial of 6 mg/kg every 3 weeks reported QTc effects. Eighty-seven patients with solid tumors were evaluated for the cardiac electrophysiologic effect of ziv-aflibercept. While no clinically significant changes were noted (defined as > 20 ms as corrected for placebo), due to limitations in the study design a small increase in QTc cannot be excluded at this time (Sanofi-Aventis, 2012).

The potential of immunogenicity was observed in patients with various cancers across 15 studies, 1.4% (41/2862) of patients tested positive for antiproduct antibody (APA) at baseline. The incidence of APA development in patients receiving ziv-aflibercept was 3.1% compared to 1.7% receiving placebo. Of those who tested positive and with sufficient samples for further testing, neutralizing antibodies were detected in 17 of 48 ziv-aflibercept patients and in 2 of 40 placebo patients (Sanofi-Aventis, 2012).

## Drug Interactions

There have been no specific drug-drug interaction studies conducted for aflibercept, although population pharmacokinetic analyses found no clinically relevant drug-drug interaction between ziv-aflibercept and irinotecan or fluorouracil (Sanofi-Aventis, 2012).

## Implications for the Future

Ziv-aflibercept is the second VEGF pathway targeted agent approved for the treatment of mCRC. Although this agent was studied in numerous solid organ malignancies, its efficacy has only been confirmed in patients with mCRC. The VELOUR study showed that the addition of ziv-aflibercept to FOLFIRI in the second-line setting resulted in improved OS and PFS. This benefit does not come without added toxicity; therefore, it is important to identify patients who would benefit most from this VEGF trap.

As new therapies are introduced, it is imperative to understand their potential role in clinical practice. Several questions remain unanswered. For example, some suggest that since ziv-aflibercept has a much higher VEGF binding affinity than bevacizumab, it could possibly be efficacious in the adjuvant setting. However, bevacizumab has failed to show benefits when combined with chemotherapy as adjuvant therapy in two large, randomized phase III trials (Wang & Lockhart, 2012).

Another area to be explored is the combination of VEGF and EGFR blockade, where again, the anti-VEGF agent bevacizumab has produced disappointing clinical data.

It should be pointed out that in terms of its current approval indication, controversy surrounded ziv-aflibercept regarding its high cost ($11,000/mo) a few months after its approval, as similar results have been obtained with bevacizumab (~1.4-month increase in OS) for half the cost (Bach, Saltz, & Wittes, 2012). In an unprecedented move, Sanofi-Aventis acknowledged market resistance and stated it would not lower the price, but it would offer discounts of up to 50% (Pollack, 2012).

## Conclusion

The treatment of mCRC remains a challenge for today’s clinicians. With the addition of promising new drugs to standard chemotherapy, we have been able to prolong the lives of patients with mCRC
